# Association between ABO blood types and sporadic pancreatic neuroendocrine tumors in the Chinese Han population

**DOI:** 10.18632/oncotarget.18592

**Published:** 2017-06-21

**Authors:** Qiwen Ben, Jun Liu, Weiyi Wang, Fang Guo, Weiyan Yao, Jie Zhong, Yaozong Yuan

**Affiliations:** ^1^ Department of Gastroenterology, Ruijin Hospital, Shanghai Jiaotong University, Shanghai, China; ^2^ Department of Integrative Medicine, Zhongshan Hospital, Fudan University, Shanghai, China; ^3^ Department of Geriatrics, Ruijin Hospital, Shanghai Jiaotong University, Shanghai, China; ^4^ Department of Endoscopy, Shanghai Eastern Hepatobiliary Surgery Hospital, Shanghai, China

**Keywords:** pancreatic neuroendocrine tumors, ABO blood type, risk factors

## Abstract

**Background:**

Although the relationship between non-O blood types and the risk of exocrine pancreatic cancer has been demonstrated, the association between ABO blood types and sporadic pancreatic neuroendocrine tumor (PNET) has not been reported thus far.

**Methods:**

This hospital-based, case-control study included 387 patients with PNET and 542 age- and sex-matched controls. Unconditional multivariable logistic regression analysis was performed to estimate the adjusted odds ratios (AORs) and 95% confidence intervals (CIs). The relationship between ABO blood types and clinicopathologic features was also analyzed.

**Results:**

After adjusting for age, sex, smoking status, alcohol drinking, and first-degree family history of any cancer, the AORs (95% CI) of functional PNET were 0.87 (0.59–1.28) for blood type A, 0.86 (0.58–1.28) for blood type B, and 0.71 (0.39–1.26) for blood type AB compared with subjects with blood type O. A similar ABO blood-type distribution was observed among cases with non-functional PNETs compared with controls. On comparing blood type B with non-B blood type, cases with non-functional PNETs had marginally higher rates of lymph node invasion (P = 0.047), distant metastasis (P = 0.044), and advanced European Neuroendocrine Tumor Society Stage (P = 0.040).

**Conclusions:**

There is no association between the ABO blood group and the development of functional and non-functional PNETs. The ABO blood types are not associated with the clinicopathologic features in patients with functional and non-functional PNETs.

## INTRODUCTION

Pancreatic neuroendocrine tumors (PNETs), arising from endocrine cells of the pancreas, comprise 1.3–2.8% of new pancreatic malignancies annually [1]. Epidemiological studies have reported that the incidence of PNETs is increasing, with the annual incidence of 0.3–0.4/100,000 in the United States and 1.01/100,000 in Japan [2, 3]. PNETs can be broadly classified into functional (F, hormone-producing) and non-functional (NF) tumors [4]. NF-PNETs account for 85% of PNETs and have a significantly worse prognosis than F-PNETs [5].

Recently, many studies have indicated the presence of a relationship between the human ABO blood-type system and carcinogenesis or progression of human tumors, specifically exocrine pancreatic tumors [6–13]. Genome-wide association studies have found that the ABO gene variability is associated with the risk of pancreatic cancer in both Western [6] and Asian [7] populations. Observational studies from our group and other investigators recently showed an association between type-O blood and a low risk of exocrine pancreatic tumors [8–10]. However, only a few studies [14–17] evaluating the role of the ABO blood type in the prevalence of NETs in multiple endocrine neoplasia type 1 (MEN1) and Von Hippel-Lindau (VHL) disease, two inherited endocrine tumor syndromes, have been published thus far. Weisbrod et al. [14] found a strong trend for association between the O blood type and pancreatic disease manifestation in patients with VHL syndrome (P = 0.047). In addition, they observed a significant association between blood type O and neuroendocrine tumors (P = 0.01) in a cohort of 105 patients with MEN1 from the US [15]. However, this result was not confirmed in two studies on patients with MEN1 [16, 17].

Although the majority of PNETs are sporadic [4], to our knowledge, no study has thus far evaluated the association between the ABO blood types and the risk of sporadic PNETs. Therefore, we conducted a large case-control study to examine the relationship between the ABO blood types and PNETs in the Chinese Han ethnic population. In addition, we assessed the correlations between ABO blood types and clinicopathologic features.

## RESULTS

### Patient characteristics

The demographics of sporadic PNETs cases and controls are shown in Table 1. A total of 387 patients with PNETs were age and sex matched with 542 control subjects. The median age at diagnosis was 49.6 (range, 16–81) years for cases and 48.4 (range, 18–78) years for controls. The ratio of men to women was 1:1.21 for cases and 1:1.22 for controls; most of the cases and controls were married (90.6% vs. 88.2, respectively) and Rh factor positive (99.0% vs. 98.8, respectively). Most of the patients and controls lived in urban areas (73.9% vs. 74.0%, respectively) and were educated up to middle or high school (54.0% and 55.0%, respectively). No statistically significant differences were found between patients and controls in terms of the abovementioned variables (P > 0.05). According to the clinical symptoms and immunostaining results, 143 (37.0%) cases were classified as non-functional PNETs and 244 (63.0%), as functional PNETs. Most (56.3%) of the functional PNETs were insulinomas, and the remaining were gastrinoma, glucagonoma, VIPoma, and pancreatic polypeptidoma.

**Table 1:** Demographics and clinical features of the study population

Variables	Cases, n = 387 (%)	Controls, n=542 (%)	P
**Age**			
Median ± SD	49.6 ± 13.3	48.4±9.5	0.134*
Range	16-81	18-79	
**Gender**			0.952**
Men	175	244	
Women	212	298	
**Marital status**			0.224**
Married	351 (90.6)	478 (88.2)	
Single	36 (9.4)	64 (11.8)	
**Rh factor**			0.915**
Positive	383 (99.0)	536 (98.8)	
Negative	4 (1.0)	6 (1.2)	
**Region**,			0.519**
Urban	286 (73.9)	401 (74.0)	
Rural	91 (26.1)	141(26.0)	
**Education levels**			0.410**
Elementary school or less	16 (4.1)	29 (5.4)	
Middle or high school	209 (54.0)	298 (55.0)	
College or higher level of education	148 (38.3)	184 (33.9)	
Missing data	14 (3.6)	31 (5.7)	
**Clinical functioning**			
Nonfunctioning	143 (37.0)		
Functioning	244 (63.0)		
Insulinoma	218 (56.3)		
Gastrinoma	10 (2.6)		
Glucagonoma	11 (2.8)		
VIPoma	5 (1.7)		
Pancreatic polypeptidoma	2 (0.5)		

Note: *, student's t test; **, Pearson's *χ*2 test

### Association between ABO blood types and the risk of F- and NF-PNET development

Because functional and non-functional tumors have evidently different clinical behavior and outcome, we conducted unconditional and multivariable logistic regression analyses restricted to F-PNET (n = 244) and NF-PNET (n = 143), respectively (Table 2). Compared with subjects with blood type O, the crude OR (95% CI) for F-PNET was 0.78 (0.53–1.14) for blood type A, 0.82 (0.56–1.21) for blood type B, and 0.68 (0.38–1.21) for blood type AB. In the multivariate logistic regression analysis, the non-statistically significant ORs did not change after adjusting for age, sex, smoking status (ever vs. never), alcohol drinking (ever vs. never), and first-degree family history of any cancer (FHC) (yes vs. no). In addition, when stratifying subjects with O vs. non-O blood types, A vs. non-A blood types, and B vs. non-B blood types, non-statistically significant AORs (95% CI) of 1.19 (0.86–1.65), 0.96 (0.67–1.35), and 0.95 (0.68–1.36) were observed.

**Table 2:** Association between ABO blood types and risk of functional- and non-functional -pancreatic neuroendocrine tumors development: univariate and multivariate logistic regression analyses

	F-PNET, n	Controls, n	AOR* (95%CI)	P
O	87	165	1 (reference)	-
A	70	169	0.87(0.59-1.28)	0.475
B	67	153	0.86(0.58-1.28)	0.455
AB	20	55	0.71(0.39-1.26)	0.241
(A vs.) Non-A	164	373	0.96(0.67-1.35)	0.830
(B vs.) Non-B	187	389	0.95(0.68-1.36)	0.795
(O vs.) Non-O	157	377	1.19(0.86-1.65)	0.296

	NF-PNET, n	Controls, n	AOR* (95%CI)	P
O	46	165	1 (reference)	
A	44	169	1.01(0.62-1.63)	0.982
B	38	153	0.96(0.58-1.60)	0.885
AB	14	55	1.18(0.58-2.38)	0.650
(A vs.) Non-A	98	373	1.00(0.66-1.51)	0.990
(B vs.) Non-B	104	389	0.94(0.61-1.45)	0.778
(O vs.) Non-O	96	377	1.02(0.67-1.50)	0.966

Note: *, Multivariate adjustments included age (continuous), gender, smoking status (ever vs. never), alcohol drinking (ever vs. never), a first degree family history of any cancer (yes vs. no).

In the analysis of NF-PNET, multivariate analyses with adjustments for risk factors (age, sex, smoking status, alcohol drinking, and first-degree FHC) showed that individual ABO blood types (A, AB, B, or O) of cases were comparable to hospital-based controls, with AOR (95% CI) of 1.01 (0.62–1.63) for blood type A, 0.96 (0.58–1.60) for blood type B, and 1.18 (0.58–2.38) for blood type AB. Similarly, non-statistically significant associations were observed for O vs. non-O blood types (1.00 [0.66–1.51]), A vs. non-A blood types (0.94 [0.61–1.45]), and B vs. non-B blood types (0.94[0.61-1.45]).

We further analyzed if the frequency distribution of ABO blood types in the F-PNET and NF-PNET cases were different from those reported in the previous two cohorts (Table 3). There was no significant difference in the ABO blood-type distribution between patients with F-PNET and those reported in the population-based controls one (P = 0.461) and two (P = 0.253). A similar frequency distribution was observed (all P > 0.05) for subjects with O vs. non-O blood types, A vs. non-A blood types, and B vs. non-B blood types. In addition, null associations were observed between ABO blood types and the risk of NF-PNET development (all P > 0.05, Table 3).

**Table 3:** Distribution of ABO blood types in patients with pancreatic neuroendocrine tumors and the two Han Chinese populations from shanghai

	F-PNET, n	Population control 1, n	P	Population control 2, n	P
O	87	12646	0.461	25100	0.253
A	70	12381		25474	
B	67	11501		22531	
AB	20	4002		7783	
(A vs.) Non-A	174	28149	0.530	55788	0.371
(B vs.) Non-B	177	29029	0.751	55414	0.619
(O vs.) Non-O	157	27884	0.134	58357	0.068

	NF-PNET, n	Population control 1, n	P	Population control 2, n	P
O	46	12646	0.977	25100	0.983
A	44	12381		25474	
B	38	11501		22531	
AB	14	4002		7783	
(A vs.) Non-A	98	28149	0.910	55788	0.926
(B vs.) Non-B	104	29029	0.328	55414	0.537
(O vs.) Non-O	96	27884	0.759	58357	0.547

Note, Pearson's χ^2^ test was used for all.

We compared individual blood-type distribution in hospital-based controls and two population-based controls (Supplementary Table 1) and found that the ABO blood-type distribution was similar between the three controls (all P > 0.05), suggesting that the representativeness of hospital-based controls was adequate.

### ABO blood types and PNET clinical characteristics

The clinicopathologic variables for PNET cases grouped by ABO blood types are shown in Table 4. For cases with F-PNET, no significant differences were found with regard to age at diagnosis, sex, tumor diameter, lymph node status, distant metastasis, WHO classification, and ENETS stage according to the ABO blood types (all P > 0.05). For cases with NF-PNET, no significant differences were found with regard to age at diagnosis, sex, tumor diameter, and WHO classification when comparing O blood type with non-O blood types and A blood type with non-A blood types (all P > 0.05). However, the prevalence of lymph node invasion (P = 0.047), distant metastasis (P = 0.044), and advanced ENETS stage (P = 0.040) was higher when comparing B blood type with non-B blood types.

**Table 4:** Clinical characteristics, ENETS staging and WHO classification of pancreatic neuroendocrine tumors cases sorted by ABO blood types

Variables	O	A	B	AB	P* O vs. non-O	P* A vs. non-A	P* B vs. non-B
**F PNET, n**	87	70	67	20			
**Age, year** (Mean ±SD)	48.7±12.6	48.8±15.1	45.2±14.2	46.1±12.2	0.395	0.388	0.116
**Gender**: M/F	28/60	26/44	27/40	10/10	0.232	0.913	0.518
**Tumor diameter, cm**							
<2/2-4/>4	62/26/0	44/26/0	48/18/1	12/7/1	0.666	0.175	0.434
**Lymph node status**							
Positive/ Negative	3/84	2/68	0/67	1/19	1.000	1.000	0.190
**Distant metastasis**							
Yes/no	1	2	0	1	1.000	0.598	0.313
**WHO classification**					0.629	0.575	0.951
NET G1	63	52	49	12			
NET G2	14	9	13	6			
NEC G3	1	2	2	1			
Missing data	9	7	2	1			
**ENETS stage at diagnosis**					1.00	1.000	0.677
**I - II**	85	68	62	19			
**III-IV**	3	2	5	1			
**NF- PNET, n**	46	44	38	14			
**Age, year** Mean ±SD	52.1±10.2	52.3±12.1	54.5±13.3	52.1±13.1	0.632	0.746	0.313
**Gender**: M/F	28/18	26/18	18/20	12/2	0.856	1.000	0.122
**Tumor diameter, cm**							
<2/2-4/>4	6/26/14	7/24/13	2/24/12	2/8/4	0.959	0.622	0.326
**Lymph node status**							
Positive/ Negative	5/41	5/39	9/29	1/13	0.608	0.451	**0.047**
**Distant metastasis at diagnosis**							
Yes/no	5/41	4/40	8/30	0/14	0.779	0.479	**0.044**
**WHO classification**					0.272	0.123	0.861
**NET G1**	23	32	21	6			
**NET G2**	14	7	11	3			
**NEC G3**	5	2	3	2			
**Missing data**	4	3	3	3			
**ENETS stage at diagnosis**					0.146	0.833	**0.040**
**I - II**	36	31	21	10			
**III - IV**	11	13	17	4			

Note: *Kruskal-Wallis test was used for age, and Pearson's χ^2^ test was used for the others.

## DISCUSSION

In this large case-control study, patients with sporadic PNETs (functional and non-functional) had a similar distribution of ABO blood types compared with hospital- and population-based controls. The ABO blood types were not associated with clinical characteristics, WHO classification, and ENETS stage for F-PNETs. However, we observed a higher prevalence of lymph node invasion, distant metastasis, and advanced ENETS stage when comparing B blood type and non-B blood types.

Most epidemiological studies have suggested a role for ABO blood groups in the development of cancers, especially for exocrine pancreatic cancer. For example, results from two large observational studies of Caucasians [8] and Chinese Han populations [10] suggested that compared with subjects with blood group O, those with non-O blood types (A, AB, or B) were more likely to develop pancreatic cancer. However, it is unclear whether there is a similar relationship between ABO blood types and neuroendocrine tumors. Few studies have evaluated this relationship in the two inherited endocrine tumor syndromes MEN1 and VHL [14–17]. Weisbrod et al [14] retrospectively reviewed 181 patients with VHL syndrome and found a strong trend for association between O blood type and manifestation of pancreatic neuroendocrine disease (P = 0.047) [14]. Subsequently, the same authors retrospectively analyzed a cohort of 105 patients with MEN1 and found that the overall distribution of blood types was significantly different from that reported in the US population (P =0.02). However, this result was not confirmed in two smaller studies of MEN1 patients [16, 17]. In the current study, we retrospectively analyzed 387 patients with sporadic PNETs and found that the ABO blood types were equally distributed in patients with sporadic PNETs compared with hospital-based controls and two population-based controls, suggesting no additional value of ABO blood types in screening and surveillance practice for identifying patients at risk for the development of sporadic PNETs. Importantly, these null risk associations were observed in both functional and non-functional PNETs and were adjusted for several potential risk factors for PNET, although no established risk factors for PNET were identified [18, 19].

The ABO gene, on chromosome 9q34, determines blood types by encoding three glycosyltransferases, i.e., A, B, and H antigens [20–23]. Phenotype O is characterized by the presence of only a protein backbone, the H antigen, and absence of A and B antigens. ABH antigens are present on key receptors, such as receptors for epidermal growth factor, integrins, cadherins, and CD44, all of which control cell proliferation, adhesion, and motility [24–27]. In addition, blood type O has both anti-A and anti-B antibodies in the serum, which act as a defense system, leading to a decreased incidence of cancer. Studies by Ichikawa *et al*. suggested that the loss of A/B glycosyltransferase in human colon carcinoma cell lines enhances malignancy [28, 29].

Despite this rationale, the current study found a significant correlation between blood type B and the higher prevalence of lymph node invasion, distant metastasis, and advanced ENETS stage for non-functional PNETs. However, this was not the case for blood types A and AB. These results for blood type B may be only due to chance, because (1) there are no clear biological mechanisms to explain the differential association of blood type B vs. blood type A with development of tumor, (2) the small number of subjects included in each subgroup, and (3) significant associations (i.e. group B and TNM staging, lymph nodes status, distant metastases) are obviously not independent from each other.

These results are in contrast with our previous report of the association between non-O blood types and advanced clinical characteristics and shorter overall survival in patients with exocrine pancreatic cancer who underwent curative surgery [10]. Several lines of evidence have suggested there is an association between ABO blood types and tumor behavior and subsequent clinical outcome in patients with various malignancies, such as cancers of the lung, renal, ovarian, colorectum, and pancreas [30–35]. With regard to the association between ABO blood types and clinical behavior in patients with PNET, only two reports [14, 15] were published for patients with MEN-1 and they showed conflicting results. One was a cohort of 105 patients with MEN1 from the US, which observed that 94% [16/17] of patients with metastatic tumors had type O blood compared to 74% [32/43] of patients with a benign tumor who had non-O blood type [15]. The other was a cohort of 71 patients with MEN1 from the Netherlands [16], which found no association between blood type O and the occurrence of metastatic disease (P = 0.30) or survival (P = 0.72). All these evidences suggested that the overall effect of ABO blood types and malignancies is complex, and further studies on this issue are warranted.

The Ruijin Hospital of Shanghai Jiaotong University is a tertiary care center that is famous for its Department of Endocrinology. Therefore, a lot of patients with complaints of endocrine disease are admitted to this hospital, resulting in a higher proportion of functional PNETs in our patient series than that reported in other studies (63.0% vs. 19.1%, respectively) [36].

To our knowledge, this is the first study to determine the association between ABO blood types and sporadic PNETs. We used two groups of controls and validated the good representativeness of our hospital controls. However, our study has several limitations. First, the study design was retrospective, which implies some potential degree of variation in collecting relevant data, such as blood-type information and tumor histopathological features. However, information on the blood types was abstracted from medical charts based on routine laboratory tests and was available for more than 85% of all patients with PNETs at our institution. Moreover, pathology slides and reports were reviewed by the study pathologist, ensuring that the diagnostic inclusion criteria were met and only patients with histologically confirmed PNETs were included. This increased the reliability of our findings. In addition, our approach of retrospective data collection was appropriate owing to the low incidence of PNETs. Second, we could not exclude the potentially confounding effects due to the absence of determined risk factors for PNETs. Third, we did not have the patients’ ABO genotype data and did not analyze ABO blood group antigen expression within the tumors. Finally, a strength and a potential limitation is that we analyzed only the Chinese Han population, and therefore, our results might not be applicable to people of all ethnicities.

In conclusion, our study showed that there is no association between the ABO blood types and the risk of developing functional and non-functional PNETs. In addition, the ABO blood types were not related to the clinical characteristics of patients with sporatic PNETs. Further investigation with larger data sets is required to confirm our findings while we continue to expand our cohort in this ongoing effort.

## MATERIALS AND METHODS

### Study population

This was an ongoing hospital-based, case-control study conducted at Ruijin Hospital, Shanghai Jiantong University, China. The study protocol was performed according to the Principles of the Declaration of Helsinki and the Ethics of the Chamber of Physicians of this Hospital. Informed consent was obtained from all patients.

**Figure 1:**
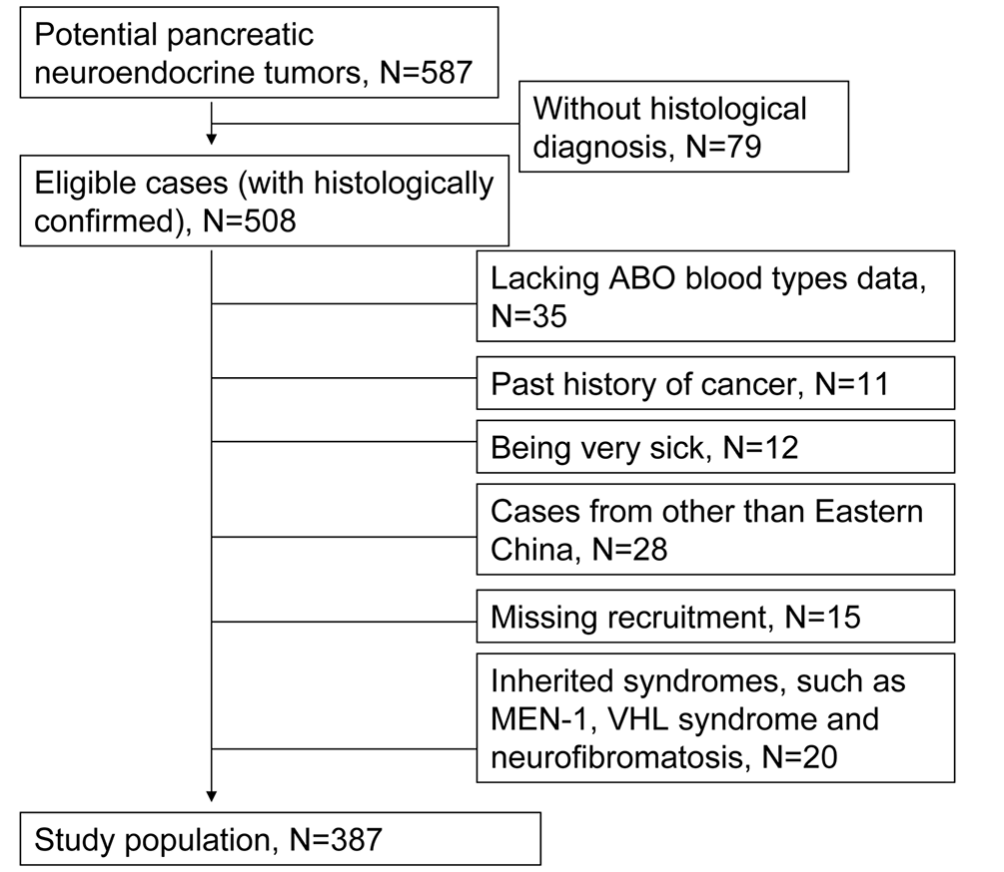
Flowchart of patient selection

Patients eligible for the study were enrolled between January 1, 2000, and June 30, 2016. Of the 587 potential PNET patients during the study period, 508 patients had histologically confirmed PNET. Of these, 35 lacked ABO blood group data, 11 had a history of cancer, 12 had a severe chronic disease (such as chronic kidney failure and heat failure) and 15 missed recruitment. Patients with a clinical diagnosis of inherited syndromes such as MEN-1, VHL, and neurofibromatosis type 1 were also excluded (n = 20). Because the distribution of the ABO blood type is associated with the geographic location, we only included patients from Eastern China (including the city of Shanghai and provinces of Jiangsu, Zhejiang, Fujian, Jiangxi, Anhui, and Shandong). Finally, a total of 387 patients were enrolled in this study (Figure 1). Eligible patients were of Chinese Han ethnicity and able to donate a blood sample at the time of diagnosis.

The diagnosis of PNET was based on conventional histology and immunohistochemistry (markers used: chromogranin A, synaptophysin, and Ki67). We also performed immunohistochemistry staining for insulin, glucagon, somatostatin, pancreatic polypeptide, gastrin, and vasoactive intestinal peptide (VIP). If the symptoms and circulating levels attributable to the corresponding peptide were concordant with immunostaining, the tumors were classified as clinically functional.

According to the intraoperative findings and pathological analysis, we recorded the tumor features such as size, lymph invasion, distant metastasis, mitotic count, and Ki-67 staining. The World Health Organization (WHO) 2010 classification was used to classify PNETs as well-differentiated endocrine tumors (G1: mitotic count, ≤2/10 high-power fields [HPF]; Ki-67, ≤2%), well-differentiated endocrine carcinomas (G2: mitotic count, 2–20/10 HPF; Ki-67, 3–20%), or poorly differentiated endocrine carcinomas (G3: mitotic count, >20/10 HPF; Ki-67, >20%) [37]. Based on the tumor-node-metastasis stage of the European Neuroendocrine Tumor Society (ENETS) [38], we stratified all PNET tumors. The test for ABO blood types and Rh factor was performed in all patients before surgery to prepare for possible bleeding and transfusion. Because the prevalence of positive Rh factor in the Chinese Han population is nearly 100%, we did not analyze the association between Rh factor and PNETs. Based on their ABO blood types, patients were stratified into three comparator groups: (1) O blood type vs. non-O blood types (A+ AB+ B), (2) A blood type vs. non-A blood types (AB+ B+ O), and (3) B blood type vs. non-B blood types (A+ AB+ O).

The first control group from the same hospital during the same period was used to examine ABO blood types distribution, as described previously [39]. Briefly, we included subjects who were diagnosed with nonmalignant disease on the basis of the discharge diagnoses. They were age- (in 3-year age groups) and sex-matched inpatients and underwent imaging and tumor marker tests (including those for CA19-9, CEA, and AFP) to exclude potential asymptomatic common tumors. Patients with a history of malignant disease or those who received any cytotoxic treatment were excluded. Patients with conditions related to alcohol and tobacco consumption (e.g., respiratory diseases, peptic ulcer, and hepatic disease) or any chronic diseases (e.g., diabetes, cardiovascular disease, and kidney disease) that might have resulted from substantial lifestyle modifications were excluded. Informed consent was obtained from all patients. After screening, we included 542 controls. The second control group included consecutive healthy blood donors from two independent population cohorts reported previously (population-based controls 1 and 2, respectively) [40, 41].

### Data collection

Cases and controls were personally interviewed for demographic characteristics (age, sex, educational level, marital status, and region), prediagnostic personal habits (smoking status and alcohol drinking), and first-degree FHC. Participants were classified as “ever-smokers” if they had smoked more than 100 cigarettes during their lifetime. Accordingly, never smokers were defined as those who smoked less than 100 cigarettes [36]. Participants were classified as “ever-drinkers” if they had consumed >1 serving/day (12.5 g/day) of alcoholic beverage (beer, wine, or liquor) for at least 6 months [42].

### Statistical analysis

Data are presented as median ± standard deviation or number (percentage), as specified. Pearson's *χ*^2^ test was performed to compare the blood-type distribution in the Chinese Han population. Crude and adjusted odds ratios (AORs) and 95% confidence intervals (CIs) for ABO blood types were calculated using unconditional logistic regression analysis. Potential confounders considered in the multivariate analysis were age (continuous), gender, smoking status, alcohol drinking, and first-degree FHC (yes vs. no). Because we were most interested in whether ABO blood types were risk factors for sporadic PNET, current smokers/drinkers and ex-smokers/drinkers were combined into a single group (ever smokers vs. never smokers; ever drinkers vs. never drinkers). Pearson's *χ*^2^ and Kruskal-Wallis test were used to evaluate the clinical characteristics in patients with F- and NF-PNET across ABO blood types. All statistical analyses were conducted using SPSS 19.0 statistical software program (SPSS, Chicago, IL, USA). Two-tailed *P* values < 0.05 were considered to be significant.

## SUPPLEMENTARY MATERIALS TABLE



## Abbreviations

PNETs: pancreatic neuroendocrine tumors
NF: non-functional
MEN1: multiple endocrine neoplasia type 1
VHL: Von Hippel-Lindau
WHO: World Health Organization
HPF: high-power fields
ENETS: European Neuroendocrine Tumor Society
FHC: family history of any cancer
AOR: adjusted odds ratio
CI: confidence interval
