# Supplementary material for: Association between ABO blood types and sporadic pancreatic neuroendocrine tumors in the Chinese Han population
Source: Oncotarget. 2017 Jun 21;8(33):54799–808. doi: 10.18632/oncotarget.18592 (PMC5589622; doi:10.18632/oncotarget.18592)
Supplement: Supplementary file 1 [file oncotarget-08-54799-s001.pdf]

Association between ABO blood types and sporadic pancreatic neuroendocrine tumors in the Chinese Han population

SUPPLEMENTARY MATERIALS

Supplementary Table 1: Distribution of ABO blood types in hospital-based controls and the two Han Chinese population-based controls

	HB control	Population control 1		Population control 2	
	N	N	P	N	P
O	165	12646	0.975	25100	0.968
A	169	12381		25474	
B	153	11501		22531	
AB	55	4002		7783	
(A vs.) Non-A	373	28149	0.751	55788	0.933
(B vs.) Non-B	389	29029	0.940	55414	0.729
(O vs.) Non-O	377	27884	0.705	58357	0.853

HB: hospital-based
